# Exercise and Peak Bone Mass

**DOI:** 10.1007/s11914-020-00588-1

**Published:** 2020-04-06

**Authors:** Magnus K. Karlsson, Björn E. Rosengren

**Affiliations:** Clinical and Molecular Osteoporosis Research Unit, Department of Orthopedics and Clinical Sciences, Lund University, Skåne University Hospital, SE – 205 02 Malmö, Sweden

**Keywords:** Bone mineral density, Children, Exercise, Muscle strength, Physical activity, Peak bone mass

## Abstract

**Purpose of review:**

The main goal of this narrative review is to assess whether physical activity (PA) influences peak bone mass and fracture risk.

**Recent findings:**

Several randomized controlled trials (RCT) show that short-term PA intervention programs in childhood improve the accrual of bone mineral. There are now also long-term controlled PA intervention studies demonstrating that both boys and girls with daily school PA through puberty gain higher bone mineral content (BMC) and bone mineral density (BMD) and greater bone size than boys and girls with school PA 1–2 times/week. These benefits seem to be followed by a gradual reduction in expected fracture rates, so that in children with daily school PA, the incidence rate ratio (IRR) after 8 years is less than half that expected by age.

**Summary:**

Daily school PA from before to after puberty is associated with beneficial gains in bone traits and gradually lower relative fracture risk.

## Introduction

### The fracture problem

Since 30% of children suffer a fracture before the age of 18 [1], and as many as 50% of women and 22% of men after the age of 50 [2], fractures have become a problem of considerable magnitude for modern society. Treatment and sick leave following these injuries result not only in individual suffering but also enormous costs for society [3]. In addition, due to demographic changes, the number of fractures is expected to increase [4]. We must therefore find intervention strategies that reduce the number of fractures, are affordable and accessible to all, and have no adverse side effects.

### Physical activity as modifiable risk factor for fractures

Researchers have tried to identify risk factors for fracture that can be addressed through interventions [5–8]. Among the strongest risk factors are low bone mineral density (BMD) [6] and frequent falls [9, 10], both associated with low physical activity (PA). In contrast, high PA in adulthood is associated with high BMD [11], superior neuromuscular function [12], low risk of falls [12, 13] and low fracture incidence [14–17]. High PA during growth is also associated with beneficial BMD and high peak bone mass (PBM) [18–22], superior neuromuscular function [18–21, 23] and greater muscle strength [18–21, 23], each of which decreases fracture risk [18, 19, 24, 25]. In fact, the National Osteoporosis Foundation’s position statement on peak bone mass development and lifestyle factors concluded in a 2016 systematic review, when grading evidence from A to D, that there is grade A (strong) evidence that PA is a modifiable lifestyle factor that may improve the development of BMD [26]. Furthermore, the same review concluded that there is grade B (moderately strong) evidence that PA is a modifiable lifestyle factor that may improve bone structural outcomes [26]. Increased PA therefore hypothetically seems to be an ideal intervention strategy, with the potential to improve musculoskeletal traits and reduce the number of fractures.

### Current level of physical activity in society

In spite of the compelling evidence that PA is beneficial for health [1, 2, 18, 20, 27–29], modern society has gradually adopted an increasingly sedentary lifestyle [30].

In 2010, about 28% of all adults (aged 18 years or older) globally were estimated to be insufficiently physically active, with a greater proportion found in high- than in low-income countries [30]. A low level of PA has also been found in individuals aged 11–17 years, where only about 20% meet the World Health Organization (WHO) PA recommendations, with girls meeting the guidelines to a lesser extent than boys [30]. The general level of PA has also declined in both sexes over the past two decades, with girls of all ages being less active than boys [31, 32]. Children also reduce their PA with increasing age [31, 32], and girls in general more than boys [33]. These observations make the need to increase the level of PA in the community even more relevant, especially during the early years of life.

### How is mechanical load mediated?

When the skeleton becomes mechanically loaded, as with PA, osteocytes have a central role in the response process. Osteocytes have a unique ability to detect and respond to mechanical strain [34]. These cells control bone formation and resorption through the differentiation of osteoblasts and osteoclasts and by stimulating the expression of the osteoclastogenesis inhibitor osteoprotegerin [35]. The exercise-induced bone adaptation process is mediated by cellular mechanotransduction [36]. Briefly, bone tissue deforms in response to the loads (strains) placed on it in response to exercise and the resultant action of muscle(s) pulling on bone, and the mechanosensors, such as stretch-activated ion channels and integrins, which are located throughout the cells, change their original configuration [37, 38]. Such changes trigger a signaling cascade that provides an appropriate biochemical response [36], e.g., bone accretion at the site of the bone deformation.

### The activitystat theory

PA is associated with a variety of health benefits [1, 2, 18, 20, 26–29]. The desired approach would therefore be to increase the level of PA in children through intervention strategies. However, according to the *activitystat theory,* this is not possible. This theory posits that PA in children is centrally regulated and kept at a constant level [39]. Intervention strategies would, according to the theory, only provide a redistribution of PA between different periods of the day, without any net increase. Thus, the theory implies that the duration of PA in children cannot be altered by intervention [39]. A review article from 2013 concluded that there is not enough evidence to refute or accept the activitystat theory [40], and that prospective controlled studies are needed to provide data with a higher level of evidence. There are other reports that infer that PA intervention programs can actually be effective in increasing the duration of PA, but only over the short term, as children often soon become bored with the intervention and return to their pre-intervention PA level [41]. According to this reasoning, all long-term PA interventions are bound to fail.

### The Pediatric Osteoporosis Prevention study

The questions raised above have been addressed in the Pediatric Osteoporosis Prevention (POP) study, a population-based prospective controlled PA interventional study in children, with the primary aim of investigating whether daily school-based PA improves musculoskeletal development and reduces fracture risk [18–21]. The POP cohort included children from four government-funded and community-based elementary schools, all located in the same city area with similar socioeconomic status. At the start of the study in grade 1, when the children were 6–9 years of age, one school increased the amount of PA to 200 min/week, provided as daily classes of 40 min. The intervention was compulsory for all children in the school on all school days. The intervention was carried out for 9 years. The extra PA included moderate to intense activities included in the regular PA curriculum such as team sports, gymnastics, running and jumping. Children in the control schools engaged in the national standard of 60 min PA/week (1–2 lessons/week) and were also followed for 9 years.

### Could population-based interventions in childhood increase the level of PA?

Results from the POP study refute the activitystat theory, with accelerometer measurements supporting the efficacy of the intervention program in increasing habitual PA levels [42]. Accelerometer measurements in children at the age 10 ± 1 years revealed that participation in the intervention program was associated with higher total duration of PA. In fact, the only independent modifiable factor that was associated with the amount of PA was participation in the intervention program [42]. After a mean of 7 years of intervention, the intervention children were more physically active by 4.5 (2.9–6.0; 95% CI) h/week than control children, when the duration of PA was evaluated by questionnaire. Furthermore, 3 years after termination of the intervention, the children in the intervention group continued to be more physically active by 2.7 (0.8–4.7; 95% CI) h/week than those in the control group. These data imply that a PA intervention program in childhood is associated with increased duration of PA both during and after the program.

### Physical activity intervention studies

The activitystat theory was refuted in the POP study, as it was evident that it was possible to increase PA duration over the entire intervention period. Other prospective controlled PA intervention studies support this hypothesis, but most include volunteers and are short-term in duration [21, 43–47]. A few long-term studies have been published [18, 48, 49], but these have not followed children from before to after puberty. All these limitations may obscure inferences, since as much as 25–40% of adult bone mass is acquired during puberty [50], and the pre- and early pubertal periods are the periods with the greatest skeletal response to mechanical load [51]. There is an ongoing debate, therefore, on whether population-based PA intervention programs do in fact enhance PBM. It is also not known whether a moderately intense PA intervention program, at a level such that all children can participate and including a variety of activities so as to prevent children from becoming bored and dropping out of the program, is enough to improve the accrual of BMD. Furthermore, the outcome may depend on whether volunteers or children at a population-based level (as in the POP cohort) are included, the latter of which is important if the aim is to implement PA in the population as a fracture prevention strategy.

### Could population-based PA interventions in childhood improve musculoskeletal traits from a long-term perspective?

The long-term POP study evaluated whether increased PA conferred beneficial effects in bone mineral content (BMC), bone mineral density (BMD), bone size [estimated as bone area (BA)], calcaneal ultrasound speed of sound (SOS) and muscle (quadriceps flexion and extension peak torque strength). These traits were followed in 140 children from before puberty [Tanner grade 1 at a mean age of 8 ± 1 (mean ± SD) years] to after puberty (Tanner stage 5 at a mean age of 15 ± 1 years) [52]. The researchers found that the gain in musculoskeletal traits was greater in boys in the intervention than boys in the control group. This was found for lumbar spine BMC, with a mean difference of 5.3 g (95% CI 1.0–9.6; *p* = 0.02), lumbar spine BMD [mean difference 0.05 g/cm^2^ (95% CI 0.01–0.10; *p* = 0.03)], lumbar spine BA [mean difference 2.2 cm^2^ (95% CI 0.2–4.1; *p* = 0.03)] and quadriceps muscle peak torque 180 degrees [mean difference 8.2 Nm (95% CI 2.2–14.2; *p* = 0.008)]. Compared with girls in the control group, girls in the intervention group achieved higher BMC at all measured sites (*p* = 0.003–0.03), including lumbar spine BMC [mean difference 6.6 g (95% CI 2.3–10.9; *p* = 0.003)], BMD total body [mean difference 0.05 g/cm^2^ (95% CI 0.02–0.08; *p* = 0.004)], lumbar spine BMD [mean difference 0.10 g/cm^2^ (95% CI 0.04–0.16); *p* = 0.002)], lumbar spine BA [mean difference 1.9 cm^2^ (95% CI 0.2–3.6; *p* = 0.03)], femoral neck BA [mean difference 0.3 cm^2^ (95% CI 0.0–0.6; *p* = 0.03)] and SOS [mean difference 41.4 m/s (15.6–67.3; *p* = 0.003)]. Together, these results show that a population-based, moderately intense PA intervention program in childhood seems to be able to provide beneficial effects in several musculoskeletal traits, all clinically relevant surrogate endpoints for fracture. There are now also data suggesting that in addition to periods of high PA, continuous PA from an early age to PBM is an important contributing factor to achieving high PBM [53, 54]. It is also possible that the total years of mechanical load may be of importance. A recent study reported that the age at which children first start walking might influence their bone strength later in life [55]. However, when conducting PA intervention programs in children, the interventions should incorporate some form of high-intensity exercise, such as high-impact jumping with sufficient ground reaction force (GRF), in order to significantly increase bone mineralization and thereby prevent osteoporosis and fragility fracture later in life [43]. In terms of interventions, there is compelling evidence that school-based exercise interventions are time- and cost-efficient and are effective in increasing BMD and/or BMC in children and adolescents [43].

### Could population-based PA interventions in childhood reduce fracture risk?

The POP study also evaluated annual fracture risk in 1339 children in the intervention and 2195 children in the control group. The researchers ascertained incident fractures in the regional radiographic archive, from study start in grade 1 when the children were 6–9 years of age, until grade 9, when the children were 15–16 years of age [18–20]. Annual fracture incidence and incidence rate ratios (IRRs) were then calculated. During the first year of participation in the study, the fracture IRR was 1.65 (1.05–2.08; mean 95% CI) in intervention children compared with controls. As the control children became older, the fracture incidence gradually increased [18–20], as in most pediatric populations [56]. In contrast, the fracture incidence in the intervention children remained almost unchanged with increasing age. This resulted in a decrease in the IRR for the intervention group each year with daily PA. After 8 years with daily PA, the relative fracture rate was 52% lower in the intervention group than in the control group [IRR 0.48 (0.25–0.91; (mean 95% CI] [19, 20], and the same trend was found in both boys and girls [18]. Based on these findings, the researchers suggest that any PA intervention program in young children should gradually increase the activity level so that the children become accustomed to a higher level of PA (reducing the increased fracture risk during the first intervention year) [18–20], but also that daily PA has the potential to reduce the expected fracture incidence from a long-term perspective.

### Does peak bone mass really correlate with peak bone strength?

This narrative review evaluates whether PA during the period of growth has the potential to increase PBM. However, the BMD benefits achieved by exercise in childhood could not explain the entire magnitude of reduced fracture risk observed [18–20]. Given this and other observations, there is concern whether BMD could selectively identify when an individual has the strongest bone during the life span. The highest BMD value during life, determined by dual-energy X-ray absorptiometry (DXA), usually defines PBM [18–22, 57]. However, DXA is based on a two-dimensional imaging technique [58], with BMD (g/cm^2^) derived by dividing the amount of BMC (in grams) by the scanned bone area (cm^2^). Decreased BMD could therefore hypothetically be the result of either (i) decreased amount of bone mineral within an anatomic region with unchanged size, (ii) increased bone size in a region with unchanged amount of bone mineral or (iii) a combination of both [22, 57]. A reduced amount of mineral leads to a weaker skeleton, while increased bone size, according to mechanical calculation, would increase bending strength in a long bone by the fourth power or the distance from the neutral axis [59]. A decreased BMD based only on increased bone size may therefore erroneously lead to the conclusion that the individual is developing a weaker skeleton, which is a problem when estimating bone strength by DXA in a growing skeleton.

Most reports infer that hip PBM occurs at ages 16–19 years [57, 60]. From an evolutionary perspective, it seems strange that hip strength should start to decline at an age when historically the ability to survive and take care of offspring has been markedly dependent on musculoskeletal strength. This raises the question of whether PBM really correlates with peak bone strength. Researchers tested this hypothesis in the population-based cross-sectional Mr PEAK study that included 1052 men aged 18–28 years. They compared bone traits by age in 1-year intervals and reported that the highest absolute femoral neck BMD was found at age 19, with statistically significant differences between the 1-year age groups in femoral neck BMC, BMD and bone area (all *p* < 0.05). From peak bone mass, there were negative correlations between age and femoral neck BMC (*r* = −0.07; *p* < 0.05) and age and femoral neck BMD (*r* = −0.12; *p* < 0.001), and positive correlation between age and femoral neck bone area (*r* = 0.06; *p* < 0.05) [57]. The bone size actually continued to increase with age to 28 years. Due to the upper age limit in the cohort, the researchers could not state whether the periosteal expansion in the skeleton ceased during the third decade in life or continued beyond age 28 years. In fact, previous prospective reports have also found a continuous periosteal expansion in women from menopause and the following decades [59, 61]. Another study that included Mr PEAK participants used peripheral computed tomography. This study supports the view that BMD (as measured by DXA) and peak bone strength may be reached at different ages, as most bone traits at diaphyseal sites were found to be greater with higher age [62].

These findings suggest that DXA-estimated peak bone mass may not correlate with peak bone strength, and suggest that future studies that interpret BMD values in the growing skeleton should do this with care, as skeletal strength may continue to increase even though DXA-estimated BMD declines.

### Physical activity-induced long-term musculoskeletal effects

Finally, the BMD value achieved during the younger years seems to be of clinical relevance from an extended perspective as well. There are now studies demonstrating that PA-induced BMD benefits attained in childhood and adolescence are partly preserved in adulthood [14–17], accompanied by lower fracture incidence in adulthood [14–17, 63]. PBM is further estimated to determine half of the variance in BMD at age 65 [64], and a 10% increase in PBM could delay the development of osteoporosis by 13 years [65]. In summary, regular PA during growth and adolescence may increase PBM, thus possibly reducing the number of fractures in adulthood.

## Conclusions

The POP study refutes the activitystat theory and suggests that the PA intervention program may increase the duration of PA in children, also extending over the longer term beyond termination of the program. The POP study also demonstrates that the school-based PA intervention program from Tanner stage 1 to 5 results in beneficial gains in BMD and bone size in both sexes, and in boys also with benefits in acquired muscle strength. These benefits could at least partly explain the gradual reduction in fracture incidence rate ratio seen in both sexes with increased PA [18, 20]. The findings may be even more interesting when viewed from a lifetime perspective, as previous studies have reported that PA-induced BMD benefits in the younger years lead to residual BMD benefits and lower fracture incidence in adulthood [14–17]. It is now essential to continue to follow the POP and similar cohorts over an even longer perspective and to collect data with a higher level of evidence, in order to verify or refute the hypothesis that PA intervention programs during growth do improve BMD and reduce the fracture burden in adulthood.
